# Altered lactate/pyruvate ratio may be responsible for aging-associated intestinal barrier dysfunction in male rats

**DOI:** 10.1007/s10522-024-10102-0

**Published:** 2024-04-15

**Authors:** Berrin Papila, Ayla Karimova, Ilhan Onaran

**Affiliations:** 1 Department of General Surgery, Cerrahpasa Faculty of Medicine, Istanbul University-Cerrahpasa, Cerrahpasa, Fatih, 34098 Istanbul, Turkey; 2 Department of Medical Biology and Genetics, Cerrahpasa Faculty of Medicine, Istanbul University-Cerrahpasa, Istanbul, Turkey

**Keywords:** Aging, Mitochondria, Common deletion, Intestinal permeability, Glycolysis, Lactate

## Abstract

Some evidence points to a link between aging-related increased intestinal permeability and mitochondrial dysfunction in in-vivo models. Several studies have also demonstrated age-related accumulation of the of specific deletion 4834-bp of “common” mitochondrial DNA (mtDNA) in various rat tissues and suggest that this deletion may disrupt mitochondrial metabolism. The present study aimed to investigate possible associations among the mitochondrial DNA (mtDNA) common deletion, mitochondrial function, intestinal permeability, and aging in rats. The study was performed on the intestinal tissue from (24 months) and young (4 months) rats. mtDNA4834 deletion, mtDNA copy number, mitochondrial membrane potential, and ATP, lactate and pyruvate levels were analyzed in tissue samples. Zonulin and intestinal fatty acid-binding protein (I-FABP) levels were also evaluated in serum. Serum zonulin and I-FABP levels were significantly higher in 24-month-old rats than 4-month-old rats (*p* = 0.04, *p* = 0.026, respectively). There is not significant difference in mtDNA4834 copy levels was observed between the old and young intestinal tissues (*p* > 0.05). The intestinal mitochondrial DNA copy number was similar between the two age groups (*p* > 0.05). No significant difference was observed in ATP levels in the intestinal tissue lysates between old and young rats (*p* > 0.05). ATP levels in isolated mitochondria from both groups were also similar. Analysis of MMP using JC-10 in intestinal tissue mitochondria showed that mitochondrial membrane potentials (red/green ratios) were similar between the two age groups (*p* > 0.05). Pyruvate tended to be higher in the 24-month-old rat group and the L/P ratio was found to be approximately threefold lower in the intestinal tissue of the older rats compared to the younger rats (*p* < 0.002). The tissue lactate/pyruvate ratio (L/P) was three times lower in old rats than in young rats. Additionally, there were significant negative correlations between intestinal permeability parameters and L/P ratios. The intestinal tissues of aged rats are not prone to accumulate mtDNA common deletion, we suggest that this mutation does not explain the age-related increase in intestinal permeability. It seems to be more likely that altered glycolytic capacity could be a link to increased intestinal permeability with age. This observation strengthens assertions that the balance between glycolysis and mitochondrial metabolism may play a critical role in intestinal barrier functions.

## Introduction

The intestinal barrier controls digestive processes, and can also create physical and immune barriers to protect the host (Gribble and Reimann 2017; Peterson and Artis 2014). The epithelial layer, a cellular component of the barrier is completely renewed every 3–5 days as a protective mechanism against minor injuries and infections (Barker 2014). Nevertheless, many studies on different rat models showed changes in the microvascular and epithelium architecture, and villus ultrastructure in the small intestine at different ages. Furthermore, intestinal permeability increases with age were also observed in 22 to 24 months old rats (Ren et al. 2014).

Several studies have defined that mitochondrial function plays a critical role in maintaining bowel permeability, motility, and homeostasis (Berger et al. 2016; Novak and Mollen 2015). These studies showed that intestinal barrier protection is energy-dependent, as in most cellular functions, and mitochondrial dysfunction can stimulate abnormal intestinal barrier function. Intestinal barrier is exposed to the deleterious effects of various environmental and lifestyle factors, as well as endogenous causes throughout human life. Due to these effects, mitochondria in intestinal cells are heavily exposed to oxidative stress (Bhattacharyya et al. 2014). Since mitochondrial DNA (mtDNA) has no protective histone protein and its repair mechanism is limited, mitochondria are more susceptible to genome damage (Roy et al. 2022). It is known from studies with different types of tissues and cells that increased oxidative stress can lead to large deletions in the mitochondrial genome (Shu et al. 2021; Dahal and Raghavan 2021).

It has been shown that the small intestine mucosa cells exhibit a high rate of glucose usage and lactate production via the glycolytic pathway (Yang et al. 2021). The results obtained from associated studies showed that the glycolytic pathway might be much more important than the oxidative phosphorylation respiratory pathway in producing ATP in intestinal tissue (Li et al. 2022). However, accumulation of the deleted mtDNA in tissue with age may disturb metabolic homeostasis by mitochondrial dysfunction, since it has been proposed that this deletion has the potential to disrupting mitochondrial metabolism (Zhang et al. 2015).

The present study aimed to investigate possible associations among the mitochondrial DNA (mtDNA) common deletion, mitochondrial function, intestinal permeability, and aging in 24-month old rats. For this purpose, mtDNA4834 deletion, mtDNA copy number, mitochondrial membrane potential, and ATP, lactate and pyruvate levels were analyzed in tissue samples. Zonulin and intestinal fatty acid-binding protein (I-FABP) levels were also evaluated in serum samples.

## Method

### Animals

Male Wistar Albino rats of young [~ 4 months (n = 6)] and old [~ 24-month-old (n = 6)] were obtained from Istanbul University Aziz Sancar Institute of Experimental Medicine, the Department of Laboratory Animals Science. All animals were in good health and were housed under controlled environmental conditions (12-h light/dark cycles, temperature 21.5 ± 0.3 °C, relative humidity 51.3 ± 3.1%, 8–10 air changes per hour). Animals had access to water ad libitum and were fed pelleted rat food with 18–20% protein-containing pellet rat food throughout the experiment. The study protocol was approved by the Animal Research Ethics Committee of Istanbul University, Istanbul, Turkey (ethical approval no: 35980450-050.01.04).

### In-vivo procedures

After fasting for 24 h, rats were anesthetized with intraperitoneal pentobarbital (40 mg/kg) and euthanized by cardiac puncture. A ~ 4-cm incision was made below the sternum along the central line of the abdomen. Small intestine samples, defined as the part of the gastrointestinal tract between the pylorus and the ileocecal valve were collected, washed with 10 ml of phosphate-buffered saline (pH 7.4), and stored at − 80 °C.

Blood samples were centrifuged at room temperature for 10 min at 1000×*g*, and the obtained serum samples were stored at − 80 °C until analysis.

### Measurement of zonulin levels

Serum zonulin (or prehaptoglobin-2) levels were measured by a commercially available enzyme-linked immunoassay (ELISA) kit (MyBiosource, San Diego, CA, USA) following the manufacturers’ instructions.

### Measurement of intestinal fatty acid-binding protein (I-FABP) levels

Serum I-FABP levels were measured by a commercially available Rat I-FABP/FABP2 ELISA Kit (Elabscience, Bethesda, MD, USA) following the manufacturers’ instructions.

### Intestinal tissue homogenization and mitochondrial isolation

The intestinal tissue (3–5 g) was cut into small pieces < 5 mm, homogenized in ice-cold homogenizing buffer (0.25 M sucrose, 5 mM HEPES buffer, and 1 mM EDTA, pH 7.2) by sonication and centrifuged at 500×*g* for 10 min at 4 °C. Subsequently the supernatants of homogenates of intestinal cells were filtered sequentially using 40-µm and 10-µm-mesh filters on ice. After centrifugation at 9400×*g* for 10 min at 4 °C, mitochondrial pellets were collected. The process was repeated diluting the pellet with the homogenizing medium (Preble et al. 2014).

Protein concentrations in all samples were measured using the Bio-Rad DCTM Protein Assay Kit (Bio-Rad Laboratories, Mississauga, Canada).

### Analysis of the amount of mtDNA^d-loop^ and mtDNA^4834^ bp deletion by real-time PCR

The homogenized intestinal tissue stored at − 80 °C was processed for mtDNA analysis using the High Pure PCR Template Preparation Kit (Roche Diagnostics, Mannheim, Germany) and subsequently tested for the presence of a 4834 bp deletion in the mitochondrial DNA with fluorescence-based quantitative PCR, using slight modified primer sets (Kang et al. 2016). A deletion-free conservative region of 80 bp was taken as reference for total mtDNA. In amplifying DNA fragments by PCR the following primer sets were used:

1. for the total mtDNA (mtDNA^d-loop^) as an internal control,
  forward 5-GGTTCTTACTTCAGGGCCATCA-3
  reverse 5-GATTAGACCCGTTACCATCGAGAT-3
2. for mtDNA^4834^bp deletion,
  forward 5-ATTTCTTCCCAAACCTTTCCTG-3
  reverse 5-GGCGTTTGATTGGATTAATGAG-3
3. for the rats ß-actin gene,
  forward 5-GGGATGTTTGCTCCAACCAA-3
  reverse 5-GCGCTTTTGACTCAAGGATTTAA-3

The PCR products of mtDNA^d-loop^, the mtDNA^4834^ mutation and ß-actin gene yielded 80 bp, 475 bp and 268 bp amplicons, respectively. Purified PCR products were cloned into the pGEM-T Easy Vector (Promega, Madison, WI, USA) and dose-dependent plasmid-constructed (ß-actin, mtDNA^d-loop^ and mtDNA^4834^) standards were used in each run of real-time PCR. The plasmids were further confirmed by DNA sequencing. The DNA samples were subjected to real-time PCR with CFX Connect™ Real-Time PCR Detection System Bio-Rad with the following reaction procedure: 95 °C for 3 min, 40 cycles of denaturation at 95 °C for 15 s, annealing at 58 °C for 30 s, and extension at 72 °C for 40 s using the SYBR PCR Mix (A&A Biotechnology RT PCR Mix SYBR A/B/C). Real-time PCR amplifications were performed in 20 μl reaction volumes containing 20 ng DNA template and 10 nM concentration of each primer. The average threshold cycle number values were used to calculate mtDNA content and deletion load in samples. Each measurement was done two or three times and normalized against a serial dilution of the corresponding plasmid clones with a known input copy number. The quantity of each target gene in our samples was calculated according to the corresponding standard curve. The formula provided by the University of Rhode Island, Genomics and Sequencing Center was used to calculate the mtDNA copy number (available from: http://cels.uri.edu/gsc/cndna.html). The level of mtDNA^4834^ was expressed as the percentage ratio of deleted mitochondrial DNA copy number to total mtDNA. Also, the mtDNA content was expressed as percentage ratio of copy number of total mitochondrial DNA to ß-actin gene copy number.

### Measurement of ATP levels

The intestinal cell homogenates or the mitochondria lysates from the intestinal tissue were neutralized with 75 μl of ice-cold 2 M KOH, 2 mM Na_2_EDTA, and 50 mM MOPS and incubated on ice for 10 min. The precipitate was pelleted by centrifugation (10,000×*g*, 1 min at room temperature). ATP levels in the supernatant were determined using the ATP Bioluminescence Assay Kit (ATP Bioluminescence Assay Kit CLC II) according to the manufacturer’s instructions (Roche, Boehringer Mannheim, Germany). The standard curve was generated from known concentrations of ATP, and the concentrations of the samples were calculated and corrected by their total protein levels.

### Mitochondrial membrane potential assay (ΔΨm)

Mitochondrial membrane potential assay (MMP) (ΔΨm) of the mitochondrial pellets in kit assay buffer and JC-10 dye loading solution (20 µM) was assessed using a JC-10 Fluorometric Assay Kit (AAT Bioquest Inc, USA) according to the manufacturer’s protocol. Spectrofluorometric analysis and settings were as follows: 540-nm excitation, 590-nm emission to detect red-fluorescence JC-10 aggregates, and 490-nm excitation, 525-nm emission to detect green fluorescence due to the ΔΨm collapse. The ratio of fluorescence intensities on emission at 525/590 was used to determine mitochondrial membrane depolarization. Data were obtained in relative fluorescence units and corrected with total protein. The JC-10 aggregate/monomer ratio is related to ΔΨm.

### Lactate and pyruvate assays

Lactate and pyruvate levels in the intestinal cell homogenates were measured with an Amplite™ Colorimetric l-Lactate Assay Kit (AAT Bioquest, Inc., Sunnyvale, CA, USA) and a Pyruvic Acid Assay Kit (Megazyme, Bray, Ireland), respectively, following the manufacturers’ guidance. The concentrations were normalized to total protein content. For insight into the shift from oxidative phosphorylation to glycolysis, we also calculated the lactate/pyruvate (L/P) ratios (Debray et al. 2007).

### Statistical analysis

Statistical analyses were performed with GraphPad Prism software 6.0 (GraphPad Software, San Diego, CA, USA). Data were expressed as mean ± SD and a two-sided P value of 0.05 was considered statistically significant. Each continuous variable was checked for normality using the Shapiro Wilks test. Continuous variables were compared by post hoc analysis using Mann–Whitney U test for nonparametric data. The correlation coefficients and their significance between mtDNA^4834^ deletion load and various parameters were calculated using Spearman’s test.

## Results

**Fig. 1:**
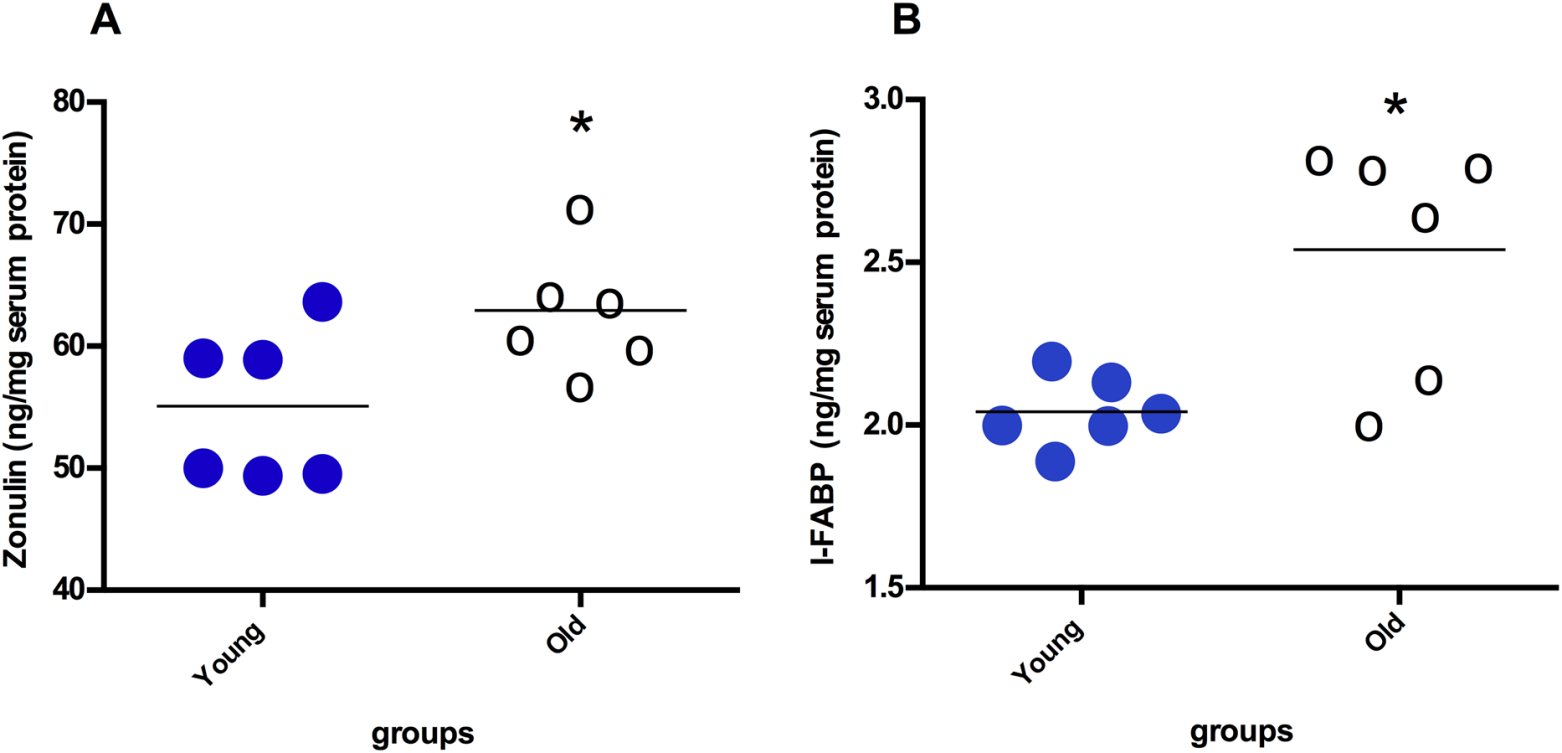
Comparison of serum zonulin (**A**) and I-FABP (**B**) levels between the 4-month-old rat group and the 24-month-old rat group. Data are expressed as the mean ± SD. * Indicates a significant difference (*p* < 0.05) compared to young rat group

Serum zonulin and I-FABP levels as intestinal permeability markers were evaluated. As shown (Fig. 1A, B), both marker levels were significantly higher in 24-month-old rats than 4-month-old rats (zonulin; 55.07 ± 6.19 ng/mg serum protein vs 62.94 ± 5.01 ng/mg serum protein in 4- and 24 month-old tissues, respectively, *p* = 0.04: I-FABP; 2.04 ± 0.11 ng/mg serum protein vs 2.54 ± 0.36 ng/mg serum protein in 4- and 24 month-old rat tissues, respectively, p = 0.026).

**Fig. 2:**
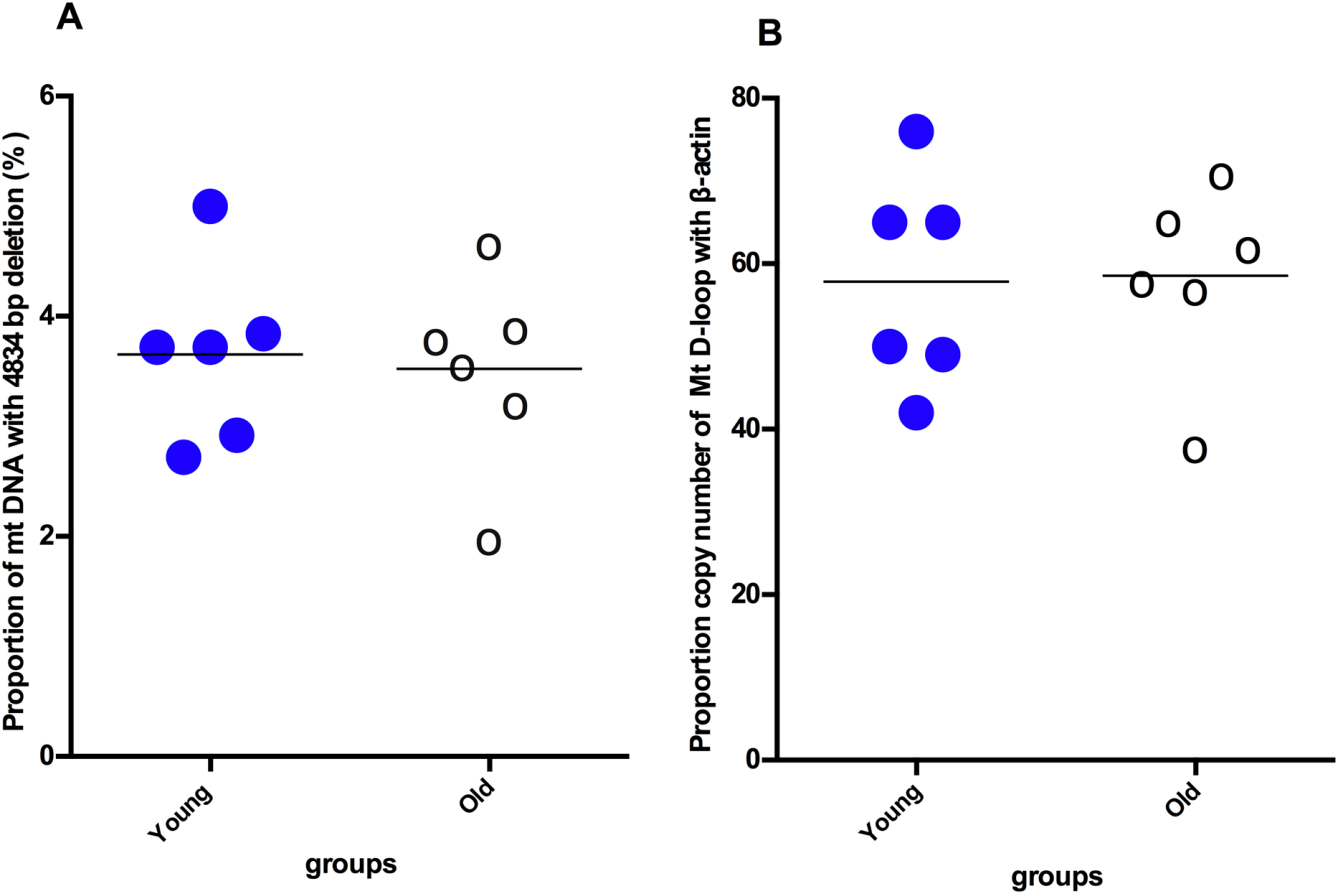
Comparison of the relative percentage of mtDNA^4834^ copy number (**A**) and mtDNA copy number (**B**) from the intestinal tissue samples of young rats (n = 6) and naturally aged rats (n = 6). Both parameters were determined by real-time PCR as described in the Methods. The percentage of deleted mtDNA was obtained from the ratio of the deleted and the conservative region of the mtDNA (representing total mtDNA-mtDNA^d-loop^) copy number measured in each sample, while mtDNA content was determined as ratio of mtDNA (mtDNA^d-loop^) to nuclear DNA (ß-actin). Data are expressed as the mean ± SD

This mutation was detected in all examined samples, however, no significant difference in mtDNA^4834^ copy levels was observed between the old and young intestinal tissues (*p* > 0.05; Fig. 2A). In addition, the intestinal mitochondrial DNA copy number was similar between the two age groups (*p* > 0.05; Fig. 2B).

**Fig. 3:**
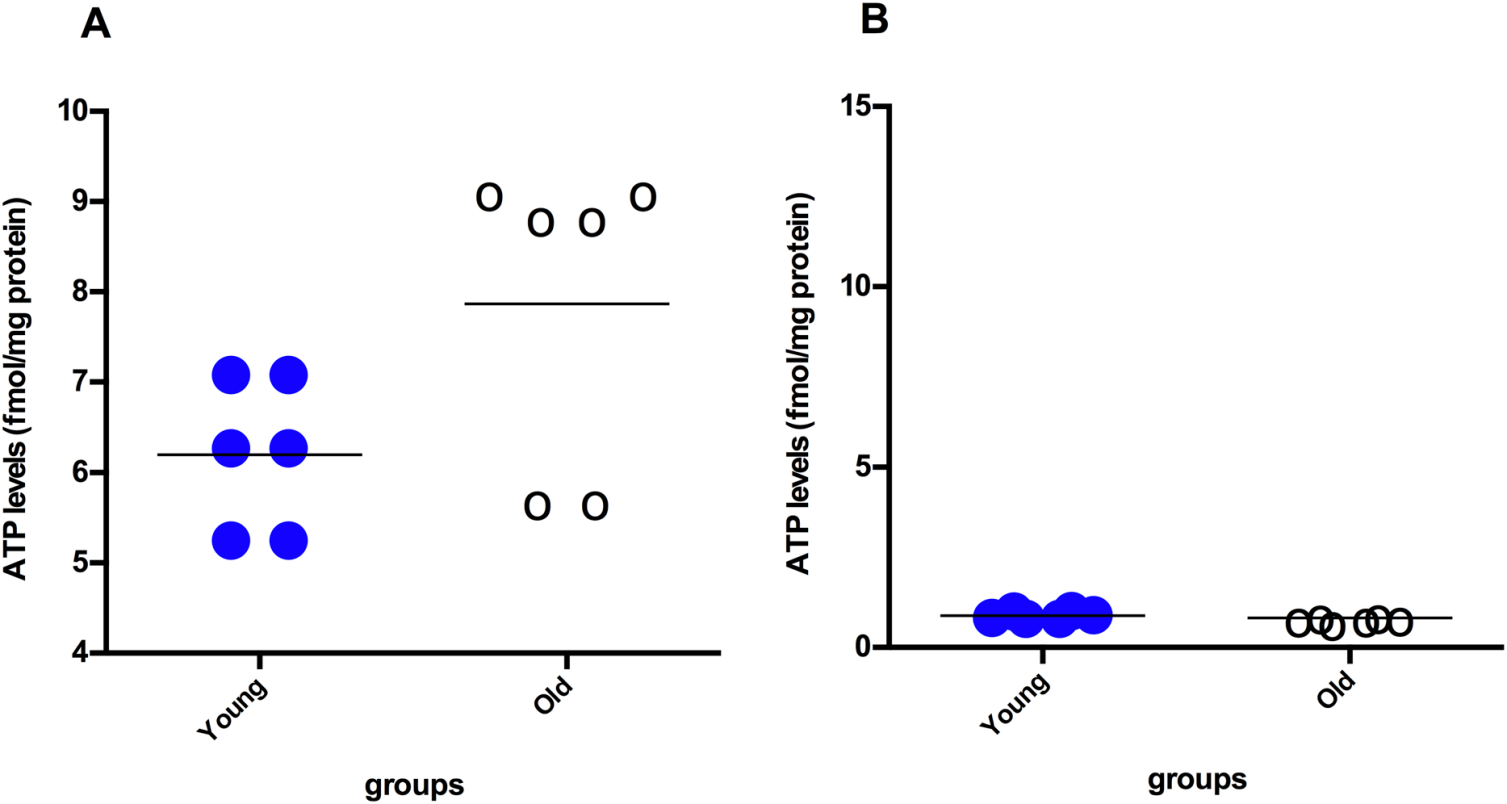
ATP levels in intestinal tissue lysates (**A**) and isolated mitochondria (**B**) from intestinal tissue samples of the 4-month-old rat (n = 6) and the 24-month-old rat (n = 6) groups. Data were analyzed with Mann–Whitney U tests and are expressed as the mean ± SD

**Fig. 4:**
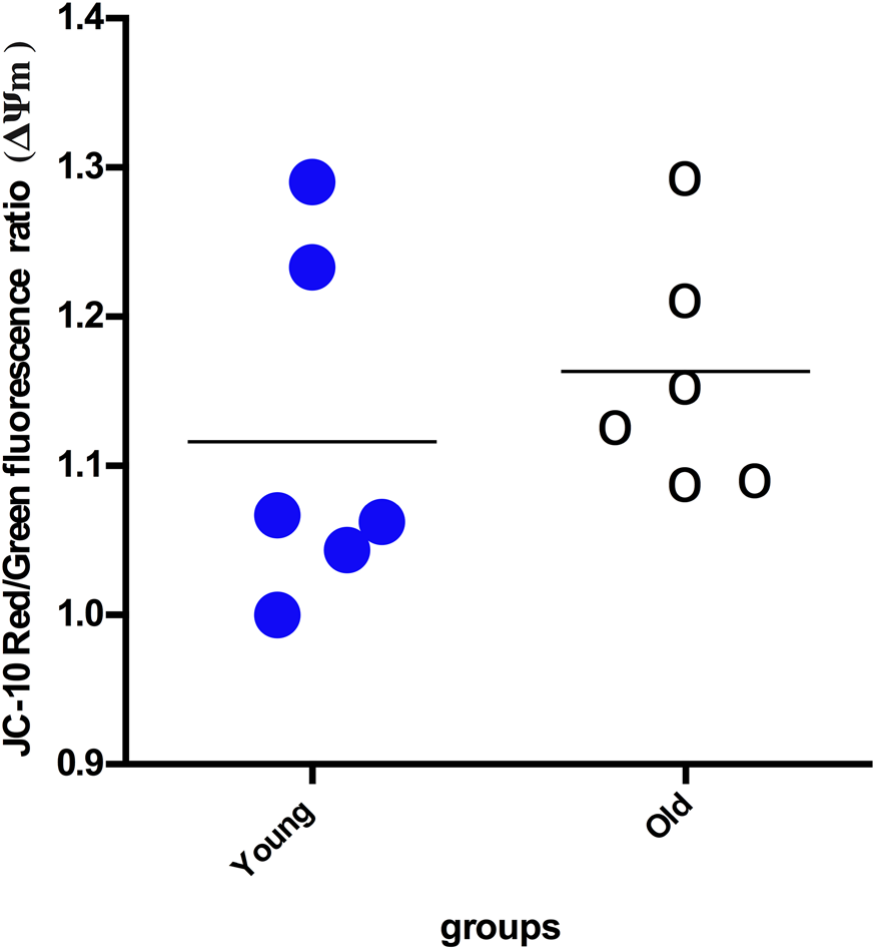
Comparison of mitochondrial membrane potential (MMP) in the mitochondria lysates from the intestinal tissue samples. MMP (ΔΨm) was determined by a JC-10 assay and data were expressed as the mean ± SD

No significant difference was observed in ATP levels in the intestinal tissue lysates between old and young rats (*p* > 0.05; Fig. 3B). Furthermore, ATP levels in isolated mitochondria from both groups were also similar (Fig. 3A). MMP was also evaluated an as indicator of mitochondrial bioenergetic function. Analysis of MMP using JC-10 in intestinal tissue mitochondria showed that mitochondrial membrane potentials (red/green ratios) were similar between the two age groups (*p* > 0.05; Fig. 4).

In this case, pyruvate tended to be higher in the 24-month-old rat group and the L/P ratio was found to be approximately threefold lower in the intestinal tissue of the older rats compared to the younger rats (*p* < 0.002; Table 1).

**Table 1:** Intracellular lactate and pyruvate levels, and lactate/pyruvate (L/P) ratio from intestinal tissue of young and aged rats

	Young rats (n = 6)	Old rats (n = 6)
Lactate (nmol/mg protein)	93.79 ± 14.79	76.57 ± 2.09*
Pyruvate (nmol/mg protein)	0.71 ± 0.15	1.74 ± 0.17*
L/P ratio	26.43 ± 2.06	8.87 ± 0.98*

*p < 0.002

From Spearman’s analyses, no significant correlations were found between the mtDNA^4834^ deletion frequency in the intestinal tissue and serum zonulin or I-FABP levels (Table 2). However, analysis of correlations between serum intestinal permeability markers and the L/P ratio in the intestinal tissue cells of rats demonstrated that both serum zonulin and I-FABP levels were negatively correlated with L/P (zonulin: r = − 0.801, *p* = 0.002 and I-FABP: r = − 0.829, *p* = 0.001; Table 2).

**Table 2:** Spearman’s correlation coefficients between intestinal permeability biomarkers and percentage mtDNA^4834^ deletion load or lactate/pyruvate in rat intestinal tissue (n = 12)

	r	*p*
Zonulin vs. mtDNA^4834^ deletion	0.248	0.433
I-FABP vs. mtDNA^4834^ deletion	0.157	0.623
Zonulin vs. Lactate/Pyruvate ratio	− 0.801	0.002*
I-FABP vs. Lactat/Pyruvate ratio	− 0.829	0.001*

*p < 0.002

## Discussion

An increased proportion of mitochondrial common DNA deletions is associated with mitochondria-related problems such as an overall decrease in energy supply and lactic acidosis. As such, it is an important factor correlated with the severity of mitochondrial disease symptoms (Zhang et al. 2015; Mohamed et al. 2006). It has been suggested that among the deletions observed in humans, mtDNA^4977^may be involved in the age-dependent decline of cell or tissue function (von Wurmb-Schwark et al. 2010; Zabihi Diba et al. 2016). In addition, mtDNA^4977^, which is considered a pathogenic mutation, may disrupt bioenergetic function by affecting the respiratory chain as previously reported (Picca et al. 2023; Peng et al. 2006). It is believed that if the ratio of deletions to wild-type mtDNA exceeds a threshold for a cell or tissue, this leads to a decrease in mitochondrial ATP production and, as a result, a decrease in the ATP/ADP ratio of associated cells. In addition, studies demonstrate that intestinal permeability is increased by the depletion of ATP, suggesting that mitochondrial energy metabolism is vital for maintaining the intestinal barrier (Schneider et al. 2020). Considering that mitochondrial dysfunction is implicated in an extensive list of various aging pathologies including intestinal barrier dysfunction (Berger et al. 2016; Tran and Greenwood-Van Meerveld 2013), this deletion may alter the metabolic properties of the intestines during aging and cause age-related changes in intestinal permeability.

Although a layer of epithelial cells forms the primary physical barrier between the lumen and mucosal tissues, intestinal organization formed by intestinal cell populations such as smooth muscle cell layers, enteric nervous system and blood vessels, as well intestinal epithelium, is essential for intestinal functions, including intestinal permeability regulation (Miguel-Aliaga et al. 2018). Previous studies reported that accumulation of the 4977-bp mtDNA deletion increases with age and may play a role in the development of aging-related pathologies (Chang et al. 2005; Zheng et al. 2012; Chen et al. 2011). mtDNA 4977 bp deletion is a common phenomenon in hair and increases with age (Zheng et al. 2012). The mtDNA 4977 bp deletion may play a role in the early stage of colorectal cancer, and it is also implicated in alteration of mtDNA content in cancer cells (Chen et al. 2011). The 4977-bp deletion accumulated during aging, we found the patients under 65 years old were more likely to carry this deletion in tumor tissues. Although 12 out of 48 (25%) of colorectal cancer patients in this younger age group were found to harbor the 4977-bp deletion in tumor tissues, only 5 of 56 (8.93%) older patients carried this deletion (Chen et al. 2011). In current study, we performed mtDNA analyses of tissue homogenates to reflect the general status of intestinal cell populations. The abundance of mtDNA^4834^ deletion did not correlate with the rat age of sampled intestinal tissue. There were no correlations between the mutation load in intestinal tissue and intestinal permeability markers.

We also assessed the bioenergetic consequences of accumulating mtDNA^4834^ deletion levels by measuring the MMP and ATP synthesis rate in intestinal tissue mitochondria. Isolation of mitochondria through tissue homogenization and differential centrifugation is ideal for studying mitochondrial bioenergetics free from the influence of other cellular factors (e.g., cytoskeleton and endoplasmic reticulum) (Brand and Nicholls 2011). In addition, use of isolated mitochondria enhances the ability to measure a fluorescence-based method for measuring mitochondrial membrane potential, because the isolation allows the mitochondria to diffuse in a small volume. Therefore, we also prefer to determine mitochondrial membrane potential in isolated mitochondria rather than whole cells. Our results showed that, in compared with young rats, the mitochondria of the intestinal tissue of old rats did not have a decrease in mitochondrial function. Several studies have speculated that the common deletion during human life may be due to tissue properties and that post-mitotic tissues accumulate mitochondrial damage faster than mitotically active tissues (Barker 2014; Preble et al. 2014). Therefore, a high turnover of the intestinal epithelium may not allow an age-dependent accumulation of this deletion. Furthermore, in an mtDNA-polymerase γ (PolgD257A) mutated mouse model, it has been shown that mtDNA mutations expand clonally, causing a mosaic pattern of the oxidative phosphorylation complex defects in aging tissues, including the small intestine. These defects have been shown to have cellular effects such as a decrease in cell proliferation, an ATP-consuming process, and an increase in the frequency of apoptosis in small intestine cells (30). Although the functional consequences of these effects have not yet been fully elucidated, Fox et al. (2012) suggest that the transition from little functional impact to mitochondrial respiratory chain dysfunction may be slightly different in various cell types of intestinal tissue as well as slightly different in various tissues. Therefore, with our experimental design, we cannot be sure whether clonally expanded mitochondrial common deletion or other mtDNA mutations in rat intestine tissue may be responsible for direct or indirect functional outcomes in the context of age-related increased intestinal permeability.

This study compared mtDNA copy number in younger and older rats, as an increased mtDNA copy number has been suggested as a possible compensatory mechanism for aging-related increases in energy demand (Clay Montier et al. 2009). However, we found no significant differences between the two groups, suggesting that mtDNA copy number status in aging intestinal tissue might not play a compensatory role for ATP tissue demands. On the other hand, glycolysis, which is the first step of the glucose oxidation process that occurs in the cytosol and produces only two ATP molecules and leads to pyruvate, is mainly involved in a wide range of biological processes. Experimental evidence demonstrates that the glycolytic pathway is much more important than the oxidative phosphorylation respiratory pathway in producing energy in intestinal epithelial cells (Yang et al. 2021). Recently, researchers have shown that glycolytic genes in various tissues are down-regulated with aging, which in turn causes a reduction of glycolysis and glucose metabolism (Ma et al. 2018). To the best of our knowledge, this association is unclear. Therefore, we also examined whether increased intestinal permeability could be, at least in part, the result of age-related inactivation of glycolysis. Since it is pointed out that monitoring the lactate levels and L/P ratio in cells helps to detect imbalances in age-related energy metabolism between aerobic glycolysis and mitochondrial oxidation (Yanase et al. 2017, 2019), we also examined this parameter in intestinal cell homogenates. We detected a marked decrease in lactate levels and, accordingly, L/P ratio in the intestinal tissue samples of aged rats and found significant negative correlations between intestinal permeability parameters and tissue L/P ratios. As total ATP levels in the intestinal cell homogenates do not change in the aged tissue, our data imply the preferential use of aerobic glycolysis to generate ATP instead of mitochondrial oxidative phosphorylation in the intestinal tissue of young rats. Therefore, an imbalance in the age-related alteration of glycolytic and oxidative capacities of intestinal tissue contribute to increased permeability. At present, the basis for the change in the L/P ratio in the intestinal tissue of aged rats is unknown.

In recent years, intestinal barrier dysfunction has emerged as an evolutionarily conserved feature of aged organisms, as reported in worms, flies, fish, rodents, and primates (Salazar et al. 2023). Moreover, aging-associated intestinal barrier dysfunction has been linked to microbial alterations, elevated immune responses, metabolic alterations, systemic health decline, and mortality (Funk et al. 2020). However, the mechanisms of age‐related increases in intestinal barrier dysfunction and how the intestinal barrier maintains tissue homeostasis upon injury remain unclear.

The roles of lactate as a metabolic feedback regulator and unique signaling molecule were considered, and the function of lactylation as a novel posttranslational modifications of proteins has been explored. Therefore, lactate is recognized to play a significant role in many physiological and pathological processes, including the regulation of energy metabolism, immunity responses, memory formation, wound healing, and tumor development. These processes are regulated by lactate as a signaling molecule or through lactylation, in addition to its role as a metabolic substrate (Salazar et al. 2023). The relationship between l-lactate and “mitochondria health” may be closely related to aging processes (Funk et al. 2020). In current study, decreased Lactate/Pyruvate ratio might be related to aging-associated intestinal barrier dysfunction.

Numerous studies have shown the beneficial effects of microbiota-derived lactate as a significant factor in inducing enterocyte hyperproliferation and intestinal stem-cell-mediated epithelial development (Okada et al. 2013; Lee et al. 2018). Although various factors such as changes in diet, medication, and lifestyles lead to changes in the intestinal microbiome, its diversity generally decreases with age (Kumar et al. 2016; Lynch and Pedersen 2016). This knowledge suggests that lactate levels measured in rat intestinal tissue are affected by bacteria-derived lactate, independent of the altered glycolytic and oxidative capacities of the intestinal tissue of aged animals. With our experimental design and the literature published to date, it is not possible to provide a sufficient explanation as to what causes the decrease in lactate levels of the intestinal tissue in aged rats and whether this decrease affects intestinal permeability. Therefore, further studies are required to understand the factors affecting intestinal permeability in association with aging.

Since ageing is primarily a progressive loss of health, the focus of interventional strategies requires a shift from the treatment and prevention of diseases to the maintenance, recovery and enhancement of health. The seven knowledge gaps (the species, sexes, bodily-systems, organs, cell types, organelles, and macromolecules) have significantly implications for both the basic research in biogerontology and for the success of interventional strategies being pursued for healthy ageing and longevity of human beings (Rattan 2024).

Our study has several limitations. First, we only used tissue samples from Wistar rats having the same genetic background born and raised in the same facility under the same dietary pattern and environmental conditions. As known, lifestyle and environmental factors may influence the mutation rate in tissues and intestinal permeability. Thus, intestinal tissue from the rat model may not reflect the characteristics of human tissue. Second, the assessment of intestinal permeability was made through blood biomarkers as an indirect measurement of epithelial barrier integrity. Another limitation was that we did not separately analyze the different parts of the intestinal tract that have different functions. Therefore, these issues could have contributed to the results obtained.

## Conclusion

Our study indicates that the intestinal tissues of aged rats are not subject to the accumulation of mtDNA common deletion. The findings also demonstrated that the deletion load in the tissue did not correlate with the increased intestinal permeability of naturally aging rats. Considering a decrease in glycolytic flux in the intestinal tissue of aging rats in our study, it seems to be more likely that altered glycolytic capacity could be a link to increased intestinal permeability with age. This observation strengthens assertions that the balance between glycolysis and mitochondrial metabolism may play a critical role in intestinal barrier functions. Further molecular studies are required to understand the factors affecting intestinal permeability in association with aging.

## Data Availability

The datasets used and/or analyzed during the current study are available from the corresponding author on reasonable request.
